# Ovarian Sensitivity Decreased Significantly in Patients With Insulin Resistance Undergoing *in vitro* Fertilization and Embryo Transfer

**DOI:** 10.3389/fphys.2021.809419

**Published:** 2022-03-11

**Authors:** Yanjun Zheng, Ye Pan, Ping Li, Zhongyuan Wang, Ze Wang, Yuhua Shi

**Affiliations:** ^1^Center for Reproductive Medicine, Cheeloo College of Medicine, Shandong University, Jinan, China; ^2^Key Laboratory of Reproductive Endocrinology of Ministry of Education, Shandong University, Jinan, China; ^3^Shandong Key Laboratory of Reproductive Medicine, Jinan, China; ^4^Shandong Provincial Clinical Research Center for Reproductive Health, Jinan, China; ^5^National Research Center for Assisted Reproductive Technology and Reproductive Genetics, Shandong University, Jinan, China; ^6^Women and Children’s Hospital, School of Medicine, Xiamen University, Xiamen, China

**Keywords:** the ovarian stimulation index, insulin resistance, polycystic ovary syndrome, controlled ovarian stimulation, *in vitro* fertilization

## Abstract

Ovarian sensitivity could affect the outcome of *in vitro* fertilization and embryo transfer (IVF-ET). The objective of this study was to explore the relationship between the ovarian sensitivity index (OSI) and traditional ovarian response makers and observe the relationship between OSI and insulin resistance (IR). The patients enrolled in this study included 131 patients with polycystic ovary syndrome (PCOS) with IR (PCOS-IR), 52 patients with PCOS without IR (PCOS-N), 164 patients with control with IR (control-IR), 133 patients with control without IR (control-N), 295 patients with IR, 184 patients with non-IR, 183 patients with PCOS, and 297 patients with control (patients with non-PCOS). All patients received standard long protocol or the gonadotropin-releasing hormone (GnRH) antagonist protocol to induce follicular development. The two protocols downregulated the pituitary function or blocked the pituitary luteinizing hormone (LH) secretion with a GnRH antagonist. Both protocols can block premature LH surges because premature luteinization is not conducive to follicular development. All patients underwent IVF or intracytoplasmic sperm injection (ICSI). Embryo transfer was carried out according to the specific situation of each patient. The OSI was significantly reduced in patients with IR. The OSI had a significant positive relationship with anti-Müllerian hormone (AMH), antral follicle count (AFC), basal LH/follicle-stimulating hormone (FSH), dominant follicle number on trigger day, retrieved oocytes, embryo number, and high-quality embryo number. OSI had a significant negative relationship with age, body mass index (BMI), basal FSH, initial dose of Gn, and total dose of Gn. The receiver operating characteristic (ROC) curve of OSI demonstrated a better accuracy in distinguishing patients with positive pregnancy and clinical pregnancy, with an area under the curve (AUC) of 0.662 (95% CI, 0.598–0.727) and 0.636 (95% CI, 0.577–0.695), respectively. Patients could get a higher rate of dominant follicle count (*p* < 0.0001) through the treatment of standard long protocol when compared with GnRH antagonist protocol. The OSI has a significant correlation with traditional ovarian response markers and could be a good predictor of positive pregnancy and clinical pregnancy for patients with IR.

## Introduction

Insulin resistance (IR) is defined as an impaired biological response to insulin stimulation of target tissues, mainly including the liver, muscle, and adipose tissue. IR impairs glucose metabolism and results in a compensatory increase in beta-cell insulin production and hyperinsulinemia (Freeman and Pennings, 2021). It is generally considered that Homeostatic Model Assessment of IR (HOMA-IR) ≥ 2.57 is IR. The adverse consequence of IR involves hyperglycemia, hypertension, dyslipidemia, visceral adiposity, hyperuricemia, elevated inflammatory markers, endothelial dysfunction, and a prothrombotic state (Brown et al., 2019; Deacon, 2019; Seong et al., 2019). In addition, the further development of IR may cause obesity, cardiovascular disease, non-alcoholic fatty liver disease, metabolic syndrome, and polycystic ovary syndrome (PCOS) (Freeman and Pennings, 2021).

Polycystic ovary syndrome is a common reproductive endocrine disease, which affected 8–13% of women of reproductive age (Teede et al., 2019). The main characters of PCOS include ovulatory dysfunction, hyperandrogenism, and polycystic ovary morphology on ultrasonography (Wei et al., 2017). For patients with PCOS, *in vitro* fertilization and embryo transfer (IVF-ET) technology is an effective method to help them get pregnant. During the IVF-ET treatment, it is important for the response of ovarian response to the controlled ovarian stimulation (COS) because the number of retrieval of oocytes was associated with the IVF-ET outcomes (Revelli et al., 2020). IR, a common complication of PCOS, is considered a key factor in the development of PCOS (Shorakae et al., 2018). A prospective observational study showed that IR not only could impair early embryo development but also reduce the implantation rate in IVF-ET (Chang et al., 2013). But there are some studies that showed that IR could impair the oocyte quality because the insulin sensitizer contributed to improved oocyte quality and increased embryonic developmental competence (Minge et al., 2008; Robker, 2008). In contrast, the ovarian reserve function of patients with PCOS is usually better (Shrikhande et al., 2020), and PCOS is also a risk factor for ovarian hyperstimulation syndrome (OHSS) (Banker and Garcia-Velasco, 2015). Therefore, it is necessary to develop an individualized COS protocol for patients with PCOS in IVF treatment.

In fact, the sensitivity of ovarian to exogenous gonadotropins [follicle-stimulating hormone (FSH), LH, hMG, or their combination] is varied from person to person (Revelli et al., 2020). Therefore, it is very important to predict the sensitivity of ovarian to exogenous gonadotropins as accurately as possible for the outcomes of COS and personalized treatment. In the current IVF treatment, the initial dose of exogenous gonadotropins is mostly based on patients, clinical parameters, such as age, body mass index (BMI), anti-Müllerian hormone (AMH), and antral follicle count (AFC). However, these clinical parameters still have certain limitations in terms of the accuracy of reflecting ovarian sensitivity (OS), and they cannot properly reflect the dynamic process of follicular growth in response to exogenous gonadotropins (Alviggi et al., 2018).

There are several prediction models used to predict the IVF-ET outcomes so far. The follicle output rate (FORT), referring to the ratio between the number of preovulatory and the pre-stimulation follicles, was positively correlated with the clinical pregnancy rate (Gallot et al., 2012). However, FORT has been shown to be negatively related to AMH, that is, patients could express a low FORT despite the presence of adequate ovarian reserve function (Genro et al., 2011). Follicle-to-oocyte index (FOI), known to be another prediction model of OS, is the ratio of the number of oocytes harvested after COS to the number of antral follicles before the start of COS [FOI = (Oocyte number/Antral follicle count) × 100]. In this study, when FOI > 50%, it is considered that the ovarian has normal sensitivity; on the contrary, FOI ≤ 50% means that the OS is not very well (Alviggi et al., 2018). The difference between FORT and FOI is that FOI could be affected by the technologies of oocyte retrieval and triggering for final oocyte maturation. So, FOI could be used alone or combined with FORT. The modified ovarian sensitivity index (OSI), MOSI [(Total number of follicles on Day 3 or Day 4 (≥6 mm)/Initial follicle-stimulating hormone dose) × 1,000], which is based on initial follicular measurements and the initial FSH dose (Camargo-Mattos et al., 2020). MOSI is mainly used to predict embryo quality and the possibility of pregnancy.

There is also a prediction model of OSI, and OSI = [(Number of retrieved oocytes/Total gonadotropin dose) × 1,000] is a maker that could reflect the potential of follicles to produce oocytes in response to exogenous gonadotropin stimulation (Revelli et al., 2020). OSI is an interesting index for patients who received IVF-ET. In addition, OSI could also predict the IVF-ET outcomes, and Huber et al. (2013) have reported that the clinical pregnancy rate is positively correlated with OSI. Compared with the OSI calculation method, the calculation methods of FOI, FORT, and MOSI all involve follicle numbers. Some studies have questioned the method of predicting OS by using follicles before oocyte retrieved and believed that antral follicle numbers could better reflect OS (Tomas et al., 1997).

In our study, we aimed to clarify the relationship between OSI and traditional ovarian response makers (i.e., age, BMI, AMH, and AFC), and at the same time, we also want to observe the relationship between OSI and IR; understand if these OSI could be a predictor index of the positive pregnancy and clinical pregnancy for patients with IR.

## Materials and Methods

### Patients

A total of 479 patients were enrolled from the Center for Reproductive Medicine, Shandong University in our study which included 131 patients with PCOS with IR (PCOS-IR), 52 patients with PCOS without IR (PCOS-N), 164 patients with control with IR (control-IR), 133 patients with control without IR (control-N), 295 patients with IR, 184 patients with non-IR, 183 patients with PCOS, and 297 patients with control (patients with non-PCOS). The patients with PCOS were diagnosed based on Rotterdam revised criteria after excluding patients with Cushing’s syndrome, congenital adrenal hyperplasia, and androgen-secreting tumors. The exclusion criteria of the two groups were age < 40 years old; BMI < 30 kg/m^2^; basal FSH level > 12 mIU/L; and systemic diseases, endometriosis, abnormal prolactin levels or thyroid function, immune diseases, recurrent abortion, and abnormal chromosomal. This study was approved by the Ethics Committee of the Reproductive Hospital Affiliated to Shandong University. Written informed consent was obtained from each patient.

### Controlled Ovarian Stimulation Protocols

These patients agreed to undergo standard long protocol or the gonadotropin-releasing hormone (GnRH) antagonist (0.1 mg/ampoule) protocol to promote follicular development. In the GnRH antagonist protocol, recombinant FSH was administered on Day 3 of the menstruation cycle at a dose of 75–225 IU per day. In standard long protocol, patients were administrated GnRH agonist (GnRH-a) in the midluteal phase of the previous cycle until the day of human chorionic gonadotropin (hCG) administration. After menstruation, when the serum estradiol (E_2_) level was lower than 50 pg/ml, gonadotropin stimulation was started with daily use of recombinant FSH (rFSH). The dose of gonadotropins was adjusted according to the follicular growth monitored by transvaginal ultrasonography and serum E_2_ concentrations.

### Oocyte Retrieval, *in vitro* Culture, Embryo Transfer, and Pregnancy Assessment

Human chorionic gonadotropin was used when at least two follicles reached 18 mm in diameter, and 36 h later, oocytes were retrieved by transvaginal ultrasound-guided follicular aspiration. All patients underwent IVF or intracytoplasmic sperm injection (ICSI) at the Center for Reproductive Medicine, Shandong University. The good quality embryos were defined as embryos developed from normally fertilized eggs with no fragmentation or not more than 1/3, no presence of multinucleation, 3–5 blastomeres at 48 h after egg retrieval, and at least 7 blastomeres by 72 h (Puissant et al., 1987).

Embryo transfer was carried out according to the specific situation of each patient. According to the quality of embryo standard: low-quality embryos have 2–7 blastomeres (fragmentation > 20%) and high-quality embryos have 8 or more blastomeres (fragmentation ≤ 20%), and we also collected the number of high-quality embryos. When plasma β-hCG > 10.0 IU/L on 14 days after embryo transfer, we considered that it was a positive pregnancy. The clinical pregnancy was diagnosed by the existence of the pregnancy sac and fetal heartbeat in the uterine cavity on 30 days after embryo transfer.

### Statistical Analysis

The OSI was calculated by applying the following formula (Huber et al., 2013): (number of retrieved oocytes/total gonadotropin dose) × 1,000. The rate of the dominant follicle was calculated by the following formula: the dominant follicle count on trigger day/the follicle count on trigger day.

We used SPSS 26.0 (SPSS, Chicago, IL, United States), GraphPad Prism 8.0 (GraphPad Software, CA, United States), and R studio for the statistical analyses. Data were reported as mean ± SD. Student’s *t*-test was used for between-group comparisons, and Spearman correlation analysis was used for analyzing linear associations. Receiver operating characteristic (ROC) curve analysis was used for assessing the efficiency of the OSI to evaluate the positive pregnancy and clinical pregnancy. A *p*-value of <0.05 was regarded as statistical significance.

## Results

### Ovarian Sensitivity Index Was Significantly Reduced in Patients With Insulin Resistance

**FIGURE 1:**
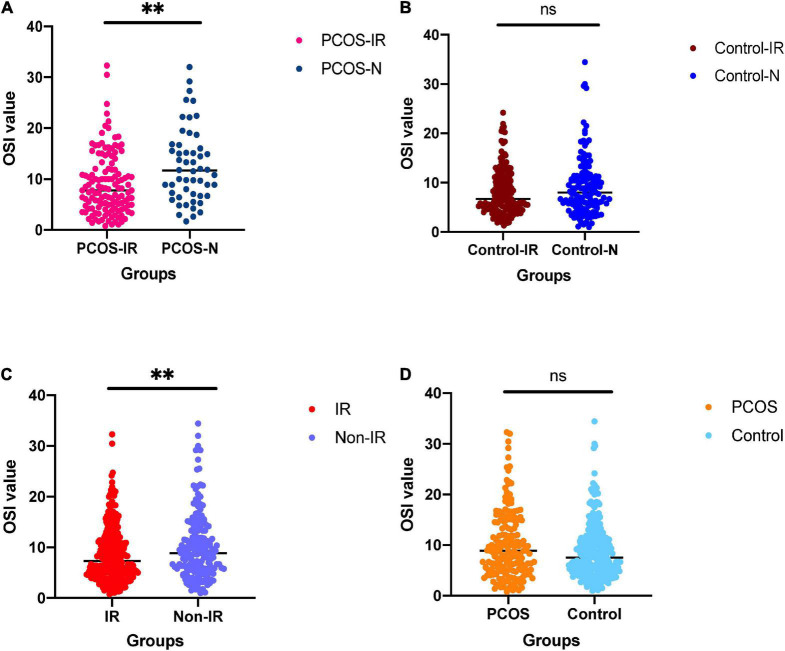
**(A)** For comparison of ovarian sensitivity index (OSI) between polycystic ovary syndrome (PCOS)-insulin resistance (IR) groups and PCOS-N group. **(B)** For comparison of OSI between the control-IR group and control-N group. **(C)** For comparison of OSI between IR group and normal group. **(D)** For comparison of OSI between PCOS group and control group. ^ns^not significant; ^∗∗^*p* < 0.01.

The clinical parameters of all patients are shown in Table 1. We compared the differences of OSI between patients with IR and patients with non-IR, patients with PCOS and patients with non-PCOS, patients with PCOS-IR and patients with PCOS-N, and patients with control-IR and patients with control-N (Table 2). The results showed that OSI decreased significantly in all patients with IR (*p* = 0.0086). Moreover, OSI also decreased significantly in patients with PCOS-IR (*p* = 0.0012). Although OSI was not significantly different between patients with control-IR and patients with control-N, OSI still has a downward trend in patients with control-IR (*p* = 0.2358). However, compared with patients with non-PCOS, OSI exhibited an increasing trend among patients with PCOS (*p* = 0.0539) (Figure 1).

**TABLE 1:** The clinical parameters of patients.

Clinical parameters	PCOS-IR	PCOS-N	Control-IR	Control-N	IR	Non-IR	PCOS	Control
Age (years)	28.91 ± 3.65	29.02 ± 3.87	30.20 ± 3.73	30.29 ± 3.15	29.62 ± 3.74	29.96 ± 3.39	28.94 ± 3.70	30.24 ± 3.47
BMI (kg/m^2^)	25.38 ± 3.04	22.43 ± 2.99	23.45 ± 3.11	21.36 ± 2.33	24.30 ± 3.22	21.64 ± 2.56	24.54 ± 3.30	22.52 ± 2.97
AMH (ng/ml)	7.66 ± 4.08	9.32 ± 4.13	3.77 ± 1.71	3.96 ± 1.96	5.46 ± 3.55	5.43 ± 3.63	8.13 ± 4.15	3.85 ± 1.83
AFC	29.77 ± 10.58	27.16 ± 6.66	16.43 ± 4.83	15.97 ± 5.59	22.39 ± 10.34	19.01 ± 7.69	29.04 ± 9.70	16.22 ± 5.18
Basal FSH (IU/L)	5.76 ± 1.48	5.75 ± 1.32	6.20 ± 1.39	6.82 ± 2.29	6.01 ± 1.45	6.50 ± 2.11	5.76 ± 1.43	6.48 ± 1.87
Basal LH (IU/L)	9.99 ± 5.98	10.65 ± 6.08	5.43 ± 3.19	6.01 ± 2.80	7.45 ± 5.16	7.26 ± 4.46	10.18 ± 6.00	5.69 ± 3.03
Basal LH/FSH	1.72 ± 0.92	1.85 ± 0.97	0.90 ± 0.49	0.91 ± 0.44	1.26 ± 0.82	1.17 ± 0.76	1.76 ± 0.93	0.90 ± 0.46
FINS	26.55 ± 20.84	8.53 ± 2.07	18.87 ± 6.59	7.54 ± 2.33	22.66 ± 15.83	7.82 ± 2.31	20.20 ± 16.33	13.11 ± 7.50
Glucose (mmol)	5.34 ± 0.43	5.17 ± 0.41	5.29 ± 0.36	5.08 ± 0.35	5.31 ± 0.39	5.10 ± 0.37	5.29 ± 0.43	5.19 ± 0.37
HOMA-IR	7.94 ± 9.51	1.93 ± 0.48	4.48 ± 1.62	1.70 ± 0.52	5.52 ± 3.82	1.76 ± 0.52	4.80 ± 3.95	3.07 ± 1.84
Initial dose of Gn (IU)	153.15 ± 33.01	134.95 ± 31.16	169.21 ± 42.48	156.06 ± 35.61	162.08 ± 39.32	150.10 ± 35.72	147.98 ± 33.44	163.34 ± 40.04
Total dose of Gn (IU)	1957.84 ± 941	1531.15 ± 813.71	1815.24 ± 701.29	1690.98 ± 641.43	1878.57 ± 818.07	1625.25 ± 637.16	1836.60 ± 924.80	1759.60 ± 676.86
Endometrial thickness on trigger day (cm)	1.10 ± 0.19	1.03 ± 0.15	1.11 ± 0.20	1.09 ± 0.18	1.10 ± 0.20	1.07 ± 0.18	1.08 ± 0.18	1.10 ± 0.19
Follicle count on trigger day	29.67 ± 7.27	30.79 ± 8.31	22.33 ± 6.07	18.37 ± 6.18	25.59 ± 7.56	17.49 ± 10.12	29.99 ± 7.58	20.56 ± 6.42
Dominant follicle count on trigger day	13.93 ± 5.00	15.06 ± 5.27	11.99 ± 4.30	12.36 ± 4.45	12.85 ± 4.72	6.03 ± 7.50	14.25 ± 5.09	12.15 ± 4.36
Retrieved oocytes	14.21 ± 6.80	16.48 ± 7.19	12.96 ± 5.36	13.22 ± 5.57	13.51 ± 6.06	14.09 ± 6.21	14.85 ± 6.97	13.07 ± 5.45
Embryo count	11.55 ± 4.53	12.83 ± 4.01	11.13 ± 3.92	11.25 ± 4.07	11.31 ± 4.20	11.70 ± 4.12	11.91 ± 4.41	11.18 ± 3.99
High-quality embryos count	5.08 ± 3.39	5.96 ± 2.70	4.50 ± 3.08	4.55 ± 3.16	4.76 ± 3.23	4.95 ± 3.10	5.33 ± 3.22	4.52 ± 3.11
OSI	9.02 ± 6.05	12.69 ± 7.28	8.29 ± 4.84	9.22 ± 6.01	8.61 ± 5.41	10.23 ± 6.56	10.06 ± 6.61	8.70 ± 5.41

*Data were presented as mean ± SD.*

**TABLE 2:** Comparison of clinical parameters between groups.

Clinical parameters	PCOS-IR vs. PCOS-N	Control-IR vs. control-N	IR vs. non-IR	PCOS vs. control
Age	0.9180	0.9624	0.3805	<0.0001
BMI	<0.0001	<0.0001	<0.0001	<0.0001
AMH	0.0089	0.5182	0.7252	<0.0001
AFC	0.3923	0.1863	0.0004	<0.0001
Basal FSH	0.6958	0.0035	0.0014	<0.0001
Basal LH	0.4575	0.0101	0.4315	<0.0001
Basal LH/FSH	0.4344	0.5467	0.2945	<0.0001
FINS	<0.0001	<0.0001	<0.0001	<0.0001
Glu	0.0055	<0.0001	<0.0001	0.0426
HOMA-IR	<0.0001	<0.0001	<0.0001	<0.0001
Initial dose of Gn	0.0001	0.0081	0.0007	<0.0001
Total dose of Gn	0.0002	0.2580	0.0016	0.5009
Endometrial thickness on trigger day	0.0630	0.2583	0.0794	0.1357
Follicle count on trigger day	0.3219	<0.0001	<0.0001	<0.0001
Dominant follicle count on trigger day	0.2367	0.6114	<0.0001	<0.0001
Retrieved oocytes	0.0241	0.5678	0.2302	0.0058
Embryo count	0.0620	0.6590	0.2584	0.0638
High-quality embryos count	0.0509	0.9732	0.4706	0.0080
OSI	0.0012	0.2358	0.0086	0.0539

### Analysis of the Correlation Between Ovarian Sensitivity Index With Clinical Parameters of Patients With Insulin Resistance

**FIGURE 2:**
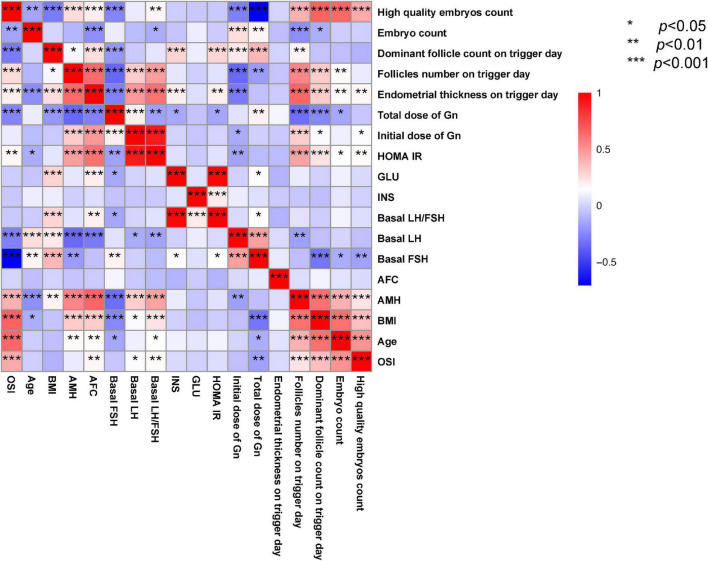
Correlation among OSI and clinical parameters of patients with IR. *p < 0.05; **p < 0.01; ***p < 0.001.

Pearson’s correlation analysis was performed between OSI with clinical parameters which included age, BMI, hormones level, endometrial thickness on trigger day, follicles situation on trigger day, embryos situation on trigger day, and some biomarkers. The results showed that OSI had a significant positive relationship with AMH, AFC, basal LH/FSH, follicle count on trigger day, dominant follicles number on trigger day, retrieved oocytes, embryos number, and high-quality embryos number. OSI had a significant negative relationship with age, BMI, basal FSH, initial dose of Gn, and total dose of Gn (Table 3 and Figure 2).

**TABLE 3:** Correlation among ovarian sensitivity index (OSI) and some clinical parameters of patients with insulin resistance (IR).

Clinical parameters	Coefficient	*p*
Age (years)	–0.1571	0.0069
BMI (kg/m^2^)	–0.2950	<0.0001
AMH (ng/ml)	0.2642	<0.0001
AFC	0.2005	0.0005
Basal FSH (IU/L)	–0.2620	<0.0001
Basal LH (IU/L)	0.07922	0.1748
Basal LH/FSH	0.1755	0.0025
FINS	–0.02196	0.7271
Glu	–0.03801	0.5155
HOMA-IR	–0.03511	0.5760
Starting dose of Gn (IU)	–0.2774	<0.0001
Total dose of Gn (IU)	–0.7101	<0.0001
Endometrial thickness on trigger day (cm)	0.001751	0.9761
Follicle count on trigger day	0.4025	<0.0001
Dominant follicle count on trigger day	0.6690	<0.0001
Retrieved oocytes	0.7897	<0.0001
Embryo count	0.6544	<0.0001
High-quality embryos count	0.4281	<0.0001

### The Relationship of Ovarian Sensitivity Index and Positive Pregnancy and Clinical Pregnancy of Patients With Insulin Resistance

**FIGURE 3:**
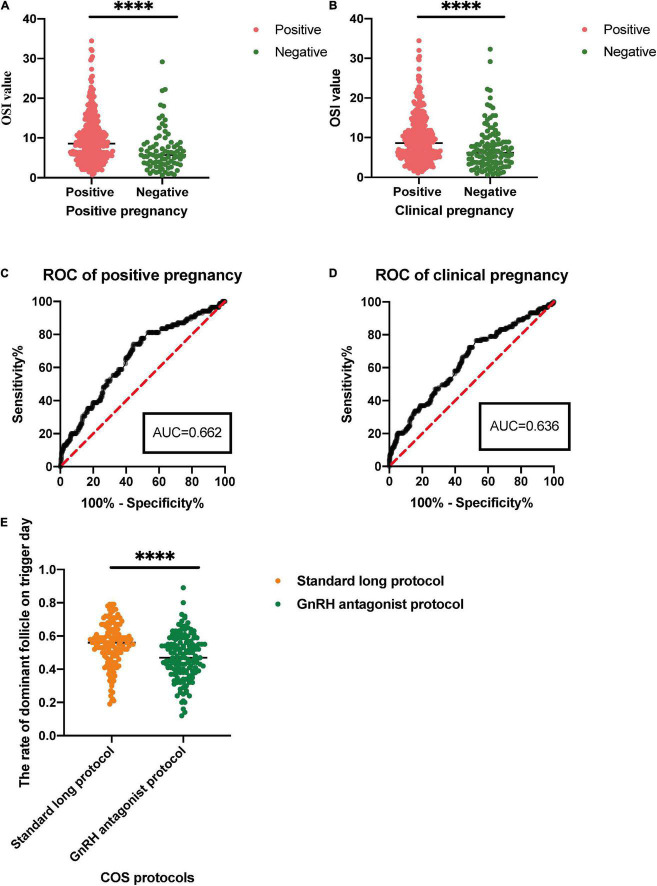
**(A,B)** For comparison of the OSI in positive pregnancy and clinical pregnancy. **(C,D)** For receiver operating characteristic (ROC) analysis of OSI to determine effectiveness in a positive pregnancy and clinical pregnancy. **(E)** For the rate of dominant follicle on trigger day in different control stimulating protocols for patients with IR. ^∗∗∗∗^*p* < 0.0001.

The OSI was significantly different between positive pregnancy patients and negative pregnancy patients (*p* < 0.0001), and it was also significantly different between clinical pregnancy patients and negative clinical pregnancy patients (*p* < 0.0001) (Table 4 and Figure 3). We used the ROC curve that analyzed the prediction efficiency for a positive pregnancy and clinical pregnancy of OSI. The results showed that the ROC curve of OSI demonstrated a better accuracy in distinguishing patients with positive pregnancy and clinical pregnancy, with an area under the curve (AUC) of 0.662 (95% CI, 0.598–0.727) and 0.636 (95% CI, 0.577–0.695), respectively (Table 5 and Figure 3).

**TABLE 4:** Comparison of the OSI in positive pregnancy and clinical pregnancy.

	Positive pregnancy	Clinical pregnancy
Positive	9.60 ± 5.86	9.67 ± 5.84
Negative	6.84 ± 5.24	7.37 ± 5.63
*t*-Test (*p*)	<0.0001	<0.0001

*Data were presented as mean ± SD.*

**TABLE 5:** Receiver operating characteristic analysis of OSI to determine effectiveness in a positive pregnancy and clinical pregnancy.

	Cut-off value	Sensitivity	Specificity	AUC (95% CI)	*p*
Positive pregnancy	7.8406	0.558	0.735	0.662 (0.598–0.727)	<0.0001
Clinical pregnancy	8.8894	0.472	0.761	0.636 (0.577–0.695)	<0.0001

### The Rate of Dominant Follicle on Trigger Day in Different Control Stimulating Protocols for Patients With Insulin Resistance

In this study, patients received standard long protocol and/or the GnRH antagonist protocol to induce follicular development, and we also compared the difference between these two COS protocols of patients with IR. The results showed that patients could get a higher rate of dominant follicle count (dominant follicle count on trigger day/all follicle counts on the trigger, *p* < 0.0001) through the treatment of standard long protocol when compared with GnRH antagonist protocol (Table 6 and Figure 3).

**TABLE 6:** The rate of dominant follicle on trigger day in different control stimulating protocols for patients with IR.

Clinical parameters	Standard long protocol	GnRH antagonist protocol	*p*
The rate of dominant follicle on trigger day	0.55 ± 0.13	0.47 ± 0.14	<0.0001
OSI	8.63 ± 4.93	8.62 ± 5.90	0.98

*Data were presented as mean ± SD.*

## Discussion

In our study, compared with patients with non-IR, the OSI in the IR group and PCOS-IR group is significantly reduced. Additionally, we observed that the OSI exhibited no significant difference between patients with PCOS and patients with control, as well as between the control-IR group and control-N. Through various cross-comparisons, we believed that IR may play an important role in reducing OS. Our results were consistent with previous reports, where the OSI is negatively correlated with age and BMI, but positively correlated with AMH and AFC (Biasoni et al., 2011). In addition, the OSI is also significantly correlated with other clinical parameters. Our results showed that the OSI is negatively correlated with the basal serum FSH level, starting dose of Gn, and total dose of Gn. In addition, the OSI is positively correlated with basal serum LH/FSH, endometrial thickness on trigger day, follicle count on trigger day, dominant follicle count on trigger day, retrieved oocytes, embryo count, and high-quality embryo count. Hyperinsulinemia could impair oocyte development competence, resulting in lower rates of fertilization, embryonic development, and implantation in patients with PCOS with obesity (Qiao and Feng, 2011). Insulin binds to receptors located on granulose cells (GCs), theca cells (TCs), and oocytes to stimulate follicle recruitment, resulting in changes in the expression of multiple genes involved in meiotic/mitotic spindle dynamics and centrosome function in PCOS oocytes (Wood et al., 2007). Dumesic et al. (2008) reported that insulin may be an important mediator of oocyte developmental competence *via* a ligand-receptor regulating system. The compensatory response to IR is hyperinsulinemia, and increased insulin could cause an increase in androgen. Excess insulin enhances androgen production in ovarian TCs under the stimulation of LH, resulting in follicular arrest and anovulation eventually (Silvestris et al., 2018). In addition, increased androgen could result in oocytes of lower quality after maturation (Cano et al., 1997). The vicious cycle of the two factors promotes the occurrence and development of PCOS. These results suggest that we need to reduce the starting dose of Gn appropriately when we encountered such patients. However, when IR occurs in patients, the starting dose of Gn or total dose of Gn should be appropriately increased considering the possibility that the OS of patients will be reduced.

Furthermore, we observed that the OSI could demonstrate a better accuracy in distinguishing patients with positive pregnancy and clinical pregnancy. With the increase of OSI, positive pregnancy and clinical pregnancy also increase. These results could be explained by the evidence that improving blastocyst and oocyte quality can increase the rate of clinical pregnancy (Zangmo et al., 2014; Wdowiak and Filip, 2020). It is suggested that the patients should reduce the insulin level before COS, which will increase the OS and improve the positive pregnancy rate and clinical pregnancy rate. Therefore, monitoring the OSI level could contribute to the judgment of positive pregnancy and clinical pregnancy. Through evaluating the patients with IR submitted to IVF who received two COS protocols, we observed that the OSI exhibited no significant difference in these two protocols, which suggests that COS protocols would not affect OSI in IVF. This result was also in line with previous study (Revelli et al., 2020). However, the rate of the dominant follicle count was significantly different between these two COS protocols for patients with IR in our study, which provided evidence that patients with IR receiving standard long protocol are more likely to harvest more dominant follicles on trigger day under the premise of clinical medication safety. Therefore, OSI could reflect the sensitivity of the ovary to Gn and could estimate the rate of positive pregnancy and clinical pregnancy for patients under standard long protocol or GnRH antagonist protocol. Compared with other markers of ovarian response, such as FOI, FORT, and MOSI, OSI could directly reflect the Gn dosage requirement of each oocyte due to its calculation method. Moreover, the OSI level was useful to determine the COS protocol and the starting dose of Gn for patients who need to receive COS for the next cycle.

Since the number of oocytes could significantly affect the probability of obtaining a live birth with a fresh embryo transfer (Sunkara et al., 2011) and the cumulative live birth rate after transferring *in utero* all thawed embryos (Fatemi et al., 2013; Li et al., 2013), harvesting more oocytes also means that patients may get a higher live birth rate. In the traditional treatment of IVF-ET, age, BMI, AFC, or AMH were often used to determine the starting dose of Gn for patients to get more oocytes. However, the factors that affect the number of oocytes are not limited to these. The choices of COS protocol, the type and dose of gonadotropins, and the ovarian inherent sensitivity of each patient should take into consideration when patients receive COS treatment. The maturation process of oocytes is very complex, in theory, the larger number of oocytes we would like to harvest, the higher level of hormonal simulation should be implemented. However, the excessive hormonal simulation would bring safety problems in clinical practice, such as OHSS (Broekmans, 2019). Therefore, the number of oocytes obtained using the traditional COS protocol may not reflect the full potential of the ovary. The OSI associates the number of oocytes with the dose of Gn, which allowing us to obtain the amount of Gn required for each oocyte to develop intuitively (Biasoni et al., 2011). Along with this perspective, we considered that the OSI was very helpful for the formulation of COS protocol and the determination of the starting dose of Gn for patients.

## Conclusion

Our study found that the OSI decreased significantly in patients with IR, and OSI has a significant correlation with traditional ovarian response markers and could be a good predictor of positive pregnancy and clinical pregnancy for patients with IR. In addition, the OSI had clinical guiding significance for patients to carry out the next cycle of COS.

## Data Availability Statement

The original contributions presented in the study are included in the article/Supplementary Material, further inquiries can be directed to the corresponding author/s.

## Ethics Statement

The studies involving human participants were reviewed and approved by the Ethics Committee of the Reproductive Hospital Affiliated to Shandong University. The patients/participants provided their written informed consent to participate in this study.

## Author Contributions

YZ and YS conceived and designed this study. ZyW collected the clinical data. YZ, YP, and ZeW contributed to statistical analysis. YZ drafted the manuscript. YZ, PL, and YS participated in the discussion and critically revised the manuscript. All authors contributed to the article and approved the submitted version.

## Conflict of Interest

The authors declare that the research was conducted in the absence of any commercial or financial relationships that could be construed as a potential conflict of interest.

## Publisher’s Note

All claims expressed in this article are solely those of the authors and do not necessarily represent those of their affiliated organizations, or those of the publisher, the editors and the reviewers. Any product that may be evaluated in this article, or claim that may be made by its manufacturer, is not guaranteed or endorsed by the publisher.
